# Gene order in rosid phylogeny, inferred from pairwise syntenies among extant genomes

**DOI:** 10.1186/1471-2105-13-S10-S9

**Published:** 2012-06-25

**Authors:** Chunfang Zheng, David Sankoff

**Affiliations:** 1 Department of Mathematics and Statistics, University of Ottawa, Canada

## Abstract

**Background:**

Ancestral gene order reconstruction for flowering plants has lagged behind developments in yeasts, insects and higher animals, because of the recency of widespread plant genome sequencing, sequencers' embargoes on public data use, paralogies due to whole genome duplication (WGD) and fractionation of undeleted duplicates, extensive paralogy from other sources, and the computational cost of existing methods.

**Results:**

We address these problems, using the gene order of four core eudicot genomes (cacao, castor bean, papaya and grapevine) that have escaped any recent WGD events, and two others (poplar and cucumber) that descend from independent WGDs, in inferring the ancestral gene order of the rosid clade and those of its main subgroups, the fabids and malvids. We improve and adapt techniques including the OMG method for extracting large, paralogy-free, multiple orthologies from conflated pairwise synteny data among the six genomes and the PATHGROUPS approach for ancestral gene order reconstruction in a given phylogeny, where some genomes may be descendants of WGD events. We use the gene order evidence to evaluate the hypothesis that the order Malpighiales belongs to the malvids rather than as traditionally assigned to the fabids.

**Conclusions:**

Gene orders of ancestral eudicot species, involving 10,000 or more genes can be reconstructed in an efficient, parsimonious and consistent way, despite paralogies due to WGD and other processes. Pairwise genomic syntenies provide appropriate input to a parameter-free procedure of multiple ortholog identification followed by gene-order reconstruction in solving instances of the "small phylogeny" problem.

## Background

Despite a tradition of inferring common genomic structure among plants, e.g., [1], and despite plant biologists' interest in detecting synteny, e.g., [2,3], the automated ancestral genome reconstruction methods developed for animals [4-7] and yeasts [8-12] at the syntenic block or gene order levels, have yet to be applied to the recently sequenced plant genomes. Reasons for this include:

1. The relative recency of these data. Although almost twenty dicotyledon angiosperms have been sequenced and released, most of this has taken place in the last two years (at the time of writing) and the comparative genomics analysis has been reserved by the various sequencing consortia for their own first publication, often delayed for years following the initial data release.

2. Algorithms maximizing a well-defined objective function for reconstructing ancestors through the median constructions and other methods are computationally costly, increasing both with *n*, the number of genes orthologous across the genomes, and especially with $\frac{d}{n}$, where *d *is the number of rearrangements occurring along a branch of the tree. Indeed, even with moderate values of $\frac{d}{n}$, these methods may fail to execute at all in reasonable time.

3. Whole genome duplication (WGD), which is rife in the plant world, particularly among the angiosperms [13,14], sets up a comparability barrier between those species descending from a WGD event and species in all other lineages originating before the event [3]. This is largely due to the process of duplicate gene reduction, eventually affecting most pairs of duplicate genes created by the WGD, which distributes the surviving members of duplicate pairs between two homeologous chromosomal segments in an unpredictable way, *fractionation *[15-19], thus scrambling gene order and disrupting the phylogenetic signal. This difficulty is compounded by the residual duplicate gene pairs created by the WGD, complicating orthology identification essential for gene order comparison between species descended from the doubling event and those outside it.

4. Global reconstruction methods are initially designed to work under the assumption of identical gene complement across the genomes, but if we look at dicotyledons, for example, each time we increase the set of genomes being studied by one, the number of genes common to the whole set is reduced by approximately $\frac{1}{3}$. Even comparing six genomes, retaining only the genes common to all six, removes 85% of the genes from each genome, almost completely spoiling the study as far as local syntenies are concerned.

Motivated in part by these issues, we have been developing an ancestral gene order reconstruction algorithm PATHGROUPS, capable of handling large plant genomes, including descendants of WGD events, as soon as they are released, using global optimization criteria, approached heuristically, but with well-understood performance properties [10,11]. The approach responds to the difficulties enumerated above as follows:

1. The software has been developed and tested with all the released and annotated dicotyledon genome sequences, even though "ethical" claims by sequencing consortia leaders discourage the publication of the results on the majority of them at this time. In this enterprise, we benefit from the up-to-date and well organized COGE platform [2,20], with its database of thousands of genome sequences and its sophisticated, user-friendly SYNMAP facility for extraction of synteny blocks.

2. PATHGROUPS aims to rapidly reconstruct ancestral genomes according to a minimum total rearrangement count (using the DCJ metric [21]) along all the branches of a phylogenetic tree. PATHGROUPS' speed is due to its heuristic approach (greedy search with look-ahead), which entails a small accuracy penalty as $\frac{d}{n}$ increases, but allows it to return a solution for values of $\frac{d}{n}$ where exact methods are no longer feasible. The implementation first produces a rapid initial solution of the "small phylogeny" problem (i.e., where the tree topology is given and the ancestral genomes are to be constructed), followed by an iterative improvement treating each ancestral node as a median problem (one unknown genome to be constructed on the basis of the three given adjacent genomes), using techniques to avoid convergence to local minima.

3. The comparability barrier erected by a WGD event is not completely impenetrable, even though gene order fractionation is further confounded by genome rearrangement events. The WGD-origin duplicate pairs remaining in the modern genome will contain much information about gene order in the ancestral diploid, immediately before WGD. The gene order information is retrievable through the method of *genome halving *[22], which is incorporated in a natural way into PATHGROUPS, where it is combined with information on single-copy genes.

4. One of the main technical contributions of this paper is the feature of PATHGROUPS that allows the genome complement of the input genomes to vary. Where the restriction to equal gene complement would lead to reconstructions involving only about 15% of the genes, the new feature allows close to 100% of the genes with orthologs in at least two genomes to appear in the reconstructions. The other key innovation we put to phylogenetic use for the first time here is our "orthologs for multiple genomes" (OMG) method for combining the genes in the synteny block sets output by SYNMAP for pairs of genomes, into orthology sets containing at most one gene from every genome in the phylogeny [23].

Both the PATHGROUPS and the OMG procedures are parameter-free. There are no thresholds or other arbitrary settings. We argue that the appropriate moment to tinker with such parameters is during the synteny block construction and not during the orthology set construction nor the ancestral genome reconstruction. A well-tuned synteny block method goes a long way to attenuate genome alignment problems due to paralogy. It is also the appropriate point to incorporate thresholds for declaring homology, since these depend on evolutionary divergence, which is specific to pairs of genomes. Finally, the natural criteria for constructing pairwise syntenies do not extend in obvious ways to three or more genomes.

## Methods

### Six eudicotyledon sequences

**Figure 1:**
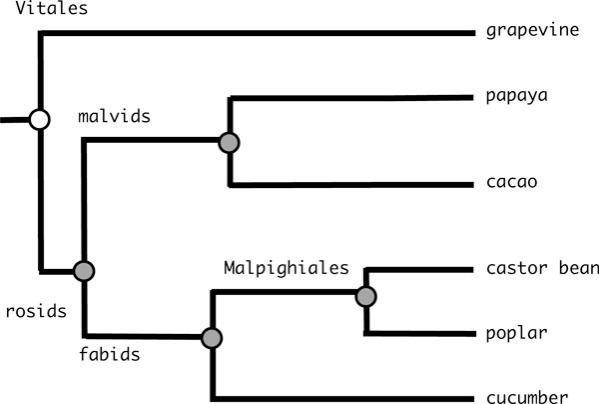
**Eudicot phylogeny**. Phylogenetic relationships among sequenced and non-embargoed eudicotyledon genomes (without regard for time scale). Poplar and cucumber each underwent WGD in their recent lineages. Shaded dots represent gene orders reconstructed here, including the rosid, fabid, malvid and Malpighiales ancestors.

There are presently almost twenty eudicotyledon genome sequences released. Removing all those that are embargoed by the sequencing consortia, all those who have undergone more than one WGD since the divergence of the eudicots from the other angiosperms, such as *Arabidopsis*, and some for which the gene annotations are not easily accessible leaves us the six depicted in Figure 1, namely cacao [24], castor bean [25], cucumber [26], grapevine [27,28], papaya [29] and poplar [30]. Of the two main eudicot clades, asterids and rosids, only the latter is represented, including the order Vitales, considered the closest relative of the eurosids [13,31]. Poplar and cucumber are the only two to have undergone ancestral WGD since the divergence of the grapevine.

### Formal methods

A genome is a set of chromosomes, each chromosome consisting of a number of genes linearly ordered. The genes are all distinct and each has positive or negative polarity, indicating on which of the two DNA strands the gene is located.

Genomes can be rearranged through the accumulated operation of number of processes: inversion, reciprocal translocation, transposition, chromosome fusion and fission. These can all be subsumed under a single operation called double-cut-and-join which we do not describe here. For our purposes all we need is a formula due to Yancopoulos *et al*. [21], stated below, that gives the genomic distance, or length of a branch in a phylogeny, in terms of the minimum number of rearrangement operations needed to transform one genome into another.

#### Rearrangement distance

**Figure 2:**
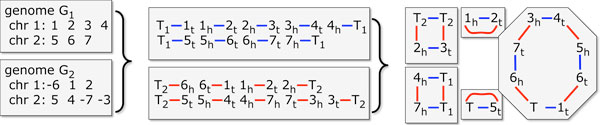
**Steps in constructing breakpoint graph**. Construction of the breakpoint graph. Left: Genomes *G*_1 _and *G*_2_, with "-" sign indicating negative polarity. Middle: Vertices and edges of individual genome graphs. Right: Cycles in completed breakpoint graph. Adapted from [10], Figure 1.

The genomic distance *d*(*G*_1_, *G*_2_) is a metric counting the number of rearrangement operations necessary to transform one multichromosomal gene order *G*_1 _into another *G*_2_, where both contain the same *n *genes. To calculate *d *efficiently, we use the breakpoint graph of *G*_1 _and *G*_2_, constructed as illustrated in Figure 2: For each genome, each gene *g *with a positive polarity is replaced by two vertices representing its two ends, i.e., by a "tail" vertex and a "head" vertex in the order *g_t_*, *g_h_*; for -*g *we would put *g_h_*, *g_t_*. Each pair of successive genes in the gene order defines an adjacency, namely the pair of vertices that are adjacent in the vertex order thus induced. For example, if *i*, *j*, *-k *are three neighbouring genes on a chromosome then the unordered pairs {*i_h_*, *j_t_*} and {*j_h_*, *k_h_*} are the two adjacencies they define. There are two special vertices called telomeres for each linear chromosome, namely the first vertex from the first gene on the chromosome and the second vertex from the last gene on the chromosome.

If there are *m *genes on a chromosome, there are 2*m *vertices at this stage. As mentioned, the first and the last of these vertices are telomeres. We convert all the telomeres in genome *G*_1 _and *G*_2 _into adjacencies with additional vertices all labelled *T*_1 _or *T*_2_, respectively. The breakpoint graph has a blue edge connecting the vertices in each adjacency in *G*_1 _and a red edge for each adjacency in *G*_2_. We make a cycle of any path ending in two *T*_1 _or two *T*_2 _vertices, connecting them by a red or blue edge, respectively, while for a path ending in a *T*_1 _and a *T*_2_, we collapse them to a single vertex denoted "*T*".

Each vertex is now incident to exactly one blue and one red edge. This bicoloured graph decomposes uniquely into *κ *alternating cycles. If *n*' is the number of blue edges, then [21]:

(1) $$d\left(G_{1},\;G_{2}\right)=n^{\prime }-\kappa .$$

#### The median problem and small phylogeny problem

Let *G*_1_, *G*_2 _and *G*_3 _be three genomes on the same set of *n *genes. *The rearrangement median problem is to find a genome M such that d*(*G*_1_, *M*) + *d*(*G*_2_, *M*) + *d*(*G*_3_, *M*) *is minimal*.

**Figure 3:**
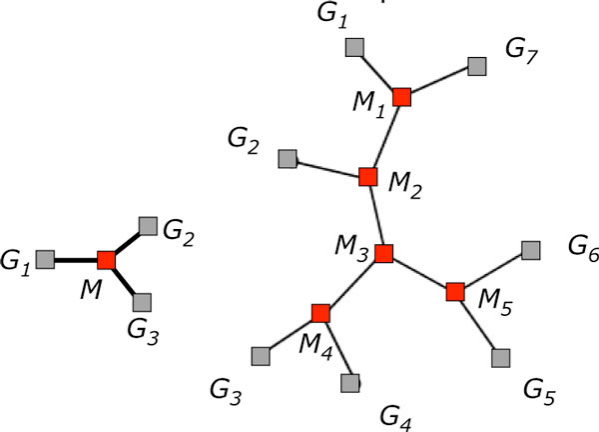
**Reconstruction problems**. Representation of median problem and small phylogeny problem. Red nodes represent ancestral genomes to be reconstructed. From [11], Figure 1.

For a given unrooted binary tree *T *on *N *given genomes *G*_1_, *G*_2_, ⋯, *G_N _*(and thus with *N *- 2 unknown ancestral genomes *M*_1_, *M*_2_, ⋯, *M*_*N *- 2 _and 2*N *- 3 branches) as depicted in Figure 3, *the small phylogeny problem is to infer the ancestral genomes so that the total edge length of T*, *namely*

(2) $$\underset{XY\in E\left(T\right)}{\sum }d\left(X,Y\right),$$

*is minimal*.

The computational complexity of the median problem, which is just the small phylogeny problem with *N *= 3, is known to be NP-hard and hence so is that of the general small phylogeny problem.

### The OMG problem

#### Pairwise orthologies

As justified in the Introduction, we construct sets of orthologous genes across the set of genomes by first identifying pairwise synteny blocks of genes. In our study, genomic data were obtained and homologies identified within synteny blocks, using the SYNMAP tool in COGE[2,20]. This was applied to the six dicot genomes in COGE shown in Figure 1, i.e., to 15 pairs of genomes. We repeated all the analyses to be described here using the default parameters of SYNMAP, with minimum block size 1, 2, 3 and 5 genes.

#### Multi-genome orthology sets

The pairwise homologies SYNMAP provided for all 15 pairs of genomes determine the set of edges *E *of the *homology graph H = (V*, *E)*, where *V *is the set of genes in any of the genomes participating in at least one homology relation.

The understanding of orthologous genes in two genomes as originating in a single gene in the most recent common ancestor of the two species, leads logically to transitivity as a necessary consequence. If gene *x *in genome *X *is orthologous both to gene *y *in genome *Y *and gene *z *in genome *Z*, then *y *and *z *must also be orthologous, even if SYNMAP does not detect any homology between *y *and *z*. The operational criteria for identifying homologs in SYNMAP, combining sequence similarity and syntenic context correspondences, may sometimes indicate that *x *is homologous to *y *and *z*, but not necessarily that *y *and *z *are homologous. This may be due to threshold criteria, differing rates or durations of evolution, or simply statistical fluctuation. Nevertheless, it seems logical to extend all homology relations by transitivity, so that in this example we will consider *y *to be homologous to *z*.

Ideally, then, all the genes in a connected component of *H *should be orthologous; insofar as SYNMAP resolves all relations of paralogy, we should expect *at most *one gene from each genome in such an orthology set, or two for genomes that descend from a WGD event.

In practice, gene *x *in genome *X *may be identified as homologous to both *y*_1 _and *y*_2 _in genome *Y*. Or *x *in *X *is homologous both to gene *y*_1 _in genome *Y *and gene *z *in genome *Z*, while *z *is also homologous to *y*_2_. By transitivity, we again obtain that *x *is homologous to both *y*_1 _and *y*_2 _in the same genome. While one gene being homologous to several paralogs in another genome is commonplace and meaningful, this should be relatively rare in the output from SYNMAP, where syntenic correspondence is a criterion for resolving paralogy. Aside from tandem duplicates, which do not interfere with gene order calculations, and duplicates stemming from WGD events (which are handled separately by our methods [10]), we consider duplicate homologs in the same genome, inferred directly by SYNMAP or indirectly by being members of the same connected component, as evidence of error or noise.

Suppose *G *= (*V_G_*, *E_G_*) is a connected component of *H *with duplicate homologs in the same genome (or more than two in the case of a WGD descendant). We delete a subset of edges *E' *⊂ *E_G_*, so that the remaining graph *Q *decomposes into smaller connected components, *Q *= *Q*_1 _∪ ⋯ ∪*Q_t_*, where each *Q_i _*is free from (non-WGD) paralogy. To decide which edges to delete, we define an objective function to be the total number of edges in the transitive closure of *Q*, i.e., in all the cliques generated by the components *Q_i_*. In other words, we seek to maximize $\sum_{1}^{t}\left(\begin{array}{c} \left|E_{i}\right|\\ 2 \end{array}\right)$, where *Q_i _*= (*V_i_*, *E_i_*). We are not aware of any algorithm for this problem, aside from the heuristic we have recently developed [23], presented here in simplified form, but conjecture it to be NP-hard.

Let $\bar{P}$ be the transitive closure of any graph *P*. To obtain $\bar{P}$ we can raise its adjacency matrix *M_P _*(including 1's on the diagonal) to successively higher powers $M_{P}^{r}$ until a maximal set of non-zero elements is attained. These non-zero elements correspond to the edges of the connectivity graph $\bar{P}$, which is the union of a set of disjoint cliques. In practice, the construction of $\bar{P}$ can be made more efficient using Warshall's algorithm [32].

The edges of  , where *G *is a component of the homology graph *H*, define a single clique, since *G *is connected. These edges represent both given and indirectly inferred orthologies as discussed above, but there may be paralogies. To remedy this by deleting edges from *G *to produce an optimal union of paralogy-free components *Q *= *Q*_1_∪ ⋯ ∪*Q_t_*, we first examine the star subgraph *s*(*v*) of  containing *ν*(*v*) vertices, namely *v*, its *ν*(*v*) *- *1 neighbours, and the *ν*(*v*) - 1 edges connecting the former to the latter.

Let *c*(*v*) ≥ 1 be the number of distinct genomes represented among the vertices in *s*(*v*). Let $F(E)=\sum_{{}_{v\in V}}c(v)$.

### Without WGD descendants

1. set *E*' = ∅.

2. **while **there are still some *v *∈ *V *where *ν*(*v*) >*c*(*v*),

(a) find the edge *e *∈ *E *\ *E*' that maximizes

$$F\left(E\backslash E^{\prime \prime }\right)=\underset{v\in V}{\sum }c\left(v\right),\;\text{where}\;E^{\prime \prime }=E^{\prime }\cup \left\{e\right\}$$

(b) **if **there are several such *e*, find the one that minimizes

$$F^{+}\left(E\backslash E^{\prime \prime }\right)=\underset{\left(V,E\backslash E^{\prime \prime }\right)}{\sum }\nu \left(v\right)-c\left(v\right).$$

(c) *E *← *E*' ∪ {*e*}

3. relabel as *Q*_1_, ⋯, *Q_t _*the disjoint components created by deleting edges. These contain the vertices of the required components of *Q*.

Implicit in each greedy step is an attempt to create large orthology sets. If the deleted edges create two partitioned components, i.e., each with no internal paralogy, then the increment in *F *will be proportional to the sum of the squares of the number of vertices in each one. This favours a decomposition into one large and one small component rather than two equal sized components.

WGD** descendants allowed**. To handle paralogs of WGD origin, the definition of *c*(*v*) must be amended to take account an allowance of 2 vertices from a single genome in *s*(*v*) if these are from the appropriate genomes. And the condition in Step 2 must require that at most two vertices be contained in *s*(*v*) from any one genome, and only if these involve WGD descendants.

Note that it is neither practical nor necessary to deal with *H *in its entirety, with its hundred thousand or so edges. It suffices to delete edges, if necessary, from each connected component *G *independently. Typically, this will contain only a few genes and very rarely more than 100. The output is a decomposition of *G *into two or more smaller sets with no undesired paralogy. These are the orthology sets we input into the gene order reconstruction step.

For the small number (typically from 1 to 5) of very large components *G *we encounter, called "tangles" in [23], we break them into more tractable size sets by extracting genes with large numbers of homologs, together with their immediate homologs, and treat them independently. This is done recursively on the remaining part of *G *until a small enough set of homologies is obtained that can be handled by the procedure detailed above.

Though these procedures require only a few minutes of computation, there are a number of devices we employ to slash this time without materially affecting the end results of our analysis. One is simply to remove at the outset all components *G *containing only two genes from two genomes separated by three or more ancestral nodes in the given phylogenetic tree. The algorithms later in our pipeline would not infer an ortholog of such genes in the ancestral genomes, so there is no point in including them in the analysis. This step allows great computational saving when the minimum size of syntenic blocks in SYNMAP is set to 1.

### PATHGROUPS

Once we have our solution to the OMG problem on the set of pairwise syntenies, we can proceed to reconstruct the ancestral genomes. First, we briefly review the PATHGROUPS approach (previously detailed in [10,11]) as it applies to the median problem with three given genomes and one ancestor to be reconstructed, *all having the same gene complement*. The same principles apply to the simultaneous reconstruction of all the ancestors in the small phylogeny problem, and to the incorporation of genomes having previously undergone WGD.

We redefine a path to be any connected subgraph of a breakpoint graph, namely any connected part of a cycle. Initially, each blue edge in the given genomes is a path. A *fragment *is any set of genes connected by red edges in a linear order. The set of fragments represents the current state of the reconstruction procedure. Initially the set of fragments contains all the genes, but no red edges, so each gene is a fragment by itself.

The objective function for the small phylogeny problem consists of the sum of a number of genomic distances, one distance for every branch in the phylogeny. Each of these distances corresponds to a breakpoint graph. A given genome determines blue edges in one breakpoint graph, while the red edges correspond to the ancestral genome being constructed. For each such ancestor, *the red edges are identical in all the breakpoint graphs corresponding to distances to that ancestor*.

A pathgroup is a set of three paths, all beginning with the same vertex, one path from each partial breakpoint graph currently being constructed. Initially, there is one pathgroup for each vertex.

**Figure 4:**
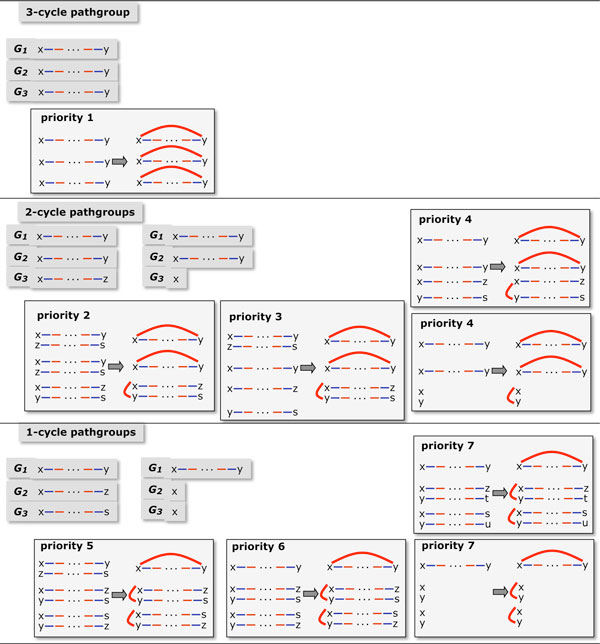
**Calculation of priorities**. Priorities of all pathgroups of form [(*x*, *a*), (*x*, *b*), (*x*, *c*)] for inserting red edges, for each ancestral vertex in the median problem. Includes sketch of three paths in "*x*" pathgroup plus other paths involved in calculating priority. For example, completing the pathgroup [(*x*, *y*), (*x*, *y*), (*x*, *z*)] by adding the red edge *xy *always produces two cycles, but can set up a pathgroup with 3 potential cycles (priority 2), 2 potential cycles (priority 3) or 1 potential cycle (priority 4). From [11], Figure 2.

Our main algorithm aims to construct three breakpoint graphs with a maximum aggregate number of cycles. At each step it adds an identical red edge to each path in the pathgroup, altering all three breakpoint graphs, as in Figure 4. It is always possible to create one cycle, at least, by adding a red edge between the two ends of any one of the paths. The strategy is to create as many cycles as possible. If alternate choices of steps create the same number of cycles, we choose one that sets up the best configuration for the next step. In the simplest formulation, the pathgroups are prioritized

1. by the maximum number of cycles that can be created within the group, without giving rise to circular chromosomes, and

2. for those pathgroups allowing equal numbers of cycles, by considering the maximum number of cycles that could be created in the next iteration of step 1, in any one pathgroup affected by the current choice.

By maintaining a list of pathgroups for each priority level, and a list of fragment endpoint pairs (initial and final), together with appropriate pointers, the algorithm requires *O*(*n*) running time.

In the current implementation of PATHGROUPS[11], much greater accuracy, with little additional computational cost, is achieved by designing a refined set of 163 priorities, based on a two-step look-ahead greedy algorithm.

For completeness, we remark that some genomes are incompletely assembled and only available in the form of fragmented chromosomes. These are treated as full chromosomes by our procedures; for this and other reasons the reconstructed ancestors may also be output as chromosomal fragments. To correct the distance between two such fragmented genomes, we note that part of the DCJ distance allows for a number of chromosomal fusions or fissions to equalize the numbers of chromosomes in the two genomes. This number is a methodological artifact and should be removed from the DCJ score to estimate the true evolutionary distance. Details of this correction have been published elsewhere [33].

#### Inferring the gene content of ancestral genomes

The assumption of equal gene content simplifies the mathematics of PATHGROUPS and allows for rapid computation. Unfortunately it also drastically reduces the number of genes available for ancestral reconstruction, so that the method loses its utility when more than a few genomes are involved.

In this section, we address the problem of assigning gene content to the ancestral genomes, a question that was avoided previously when all genomes had the same content. Then in the next section we show how to adapt PATHGROUPS to the unequal gene content median problem.

There are two natural ways to assign genes parsimoniously to ancestral genomes. One is to treat a different presence or absence status at the two ends of a branch of a phylogenetic tree as an evolutionary event, and to minimize, by dynamic programming, the number of events for each gene. However, if we have a rooted tree, it is may be more appropriate to allow any number of loss events for a gene but only one gain (innovation) event, since convergent evolution of a gene is unlikely. With real data sets, however, this rule (Dollo's principle) may be too restrictive. In our implementation, we compromise, in allowing multiple gains but when there are equally costly choices during execution of the assignment algorithm, to choose the one that attributes the gain as early in the tree as possible.

Using dynamic programming on unrooted trees, our assignment of genes to ancestors simply assures that if a gene is in at least two of the three adjacent nodes of an ancestral genome, it will be in that ancestor. If it is in less than two of the adjacent nodes, it will be absent from the ancestor.

#### Median and small phylogeny problems with unequal genomes

To generalize our construction of the three breakpoint graphs for the median problem to the case of three unequal genomes, we set up the pathgroups much as before, and we use a slightly modified priority structure. Each pathgroup, however, may have three paths, as before, or only two paths, if the initial vertex of the paths comes from a gene absent from one of the leaves. Moreover, when one or two cycles are completed by drawing a red edge, this edge must be left out of the third breakpoint graph if the corresponding gene is missing from the third genome.

The consequence of this strategy is that some of the paths in the breakpoint graph will never be completed into cycles, impeding the evaluation of the objective function (1). We could continue to search for cycles under a weakened definition, but this would be computationally costly to do in an exhaustive way, spoiling the linear run time property of the algorithm.

**Figure 5:**
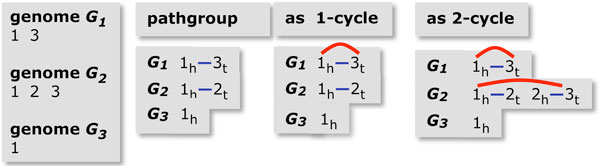
**Extension to unequal gene complements**. Handling pathgroups with unequal gene complement. Paths containing genes not in the median, such as gene 2 in the illustration, are "extended" by the sequential addition of vertices from extra genes until a vertex from a median gene in encountered. In the depicted example, this shows that there is a second, hidden, cycle involving 1*_h _*and 3*_t_*. In larger examples, this would affect the relative priority of this pathgroup. Whether or not there are hidden cycles is detected by a rapid search.

Nevertheless, we can quickly find "hidden" cycles resulting from the simple deletion of genes from one of the genomes, of an otherwise common gene sequence, a frequent occurrence. This is illustrated in Figure 5, where knowledge from a limited search can be incorporated into the priority scheme when this vertex is missing from another breakpoint graph.

The small phylogeny problem can be formulated and solved using the same principles as the median problem, as with the case of equal genomes. The solution, however, only serves as an initialization. As in [11], the solution can be improved by applying the median algorithm to each ancestral node in turn, based on the three neighbour nodes, and iterating until convergence of the total tree length (2). At each step, the new median is accepted if the sum of the three branch lengths is no greater than the existing one. This strategy of allowing the median to change as long as it does not increase total tree length is effective in exploring local solution space and avoiding local minima.

## Results

### Coping with fractionation

**Figure 6:**
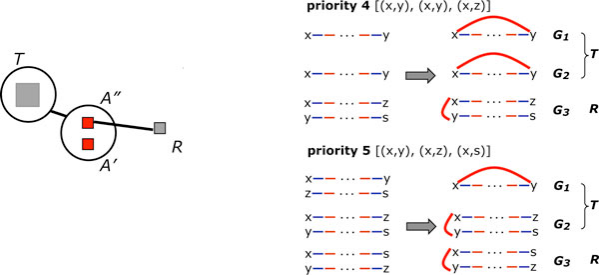
**Extension for incorporating WGD**. PATHGROUP for WGD. A consisting of two identical genomes *A' *and *A'' *on branch between descendant *T *and ancestor median *R*. Shown are are two configurations with different priorities.

As shown in Figure 6, PATHGROUPS integrates a descendant *T *of a WGD into a phylogeny by creating an immediate median-like ancestor node *A *in the tree where two of the paths (say *G*_1 _and *G*_2 _in Figure 4 connect to *T *and the third (*G*_3_) to an ancestral node *R *in the phylogeny. Like all ancestral nodes, *R *is connected to two other nodes in the tree, leaves or ancestral.

There are some technical differences connected with avoiding the creation of circular chromosomes in PATHGROUPS for WGD. Our current implementation can only handle the case where *T *contains exactly two copies of every gene in *R*. Thus we consider only the duplicate genes in *T *in constructing *A *during the small phylogeny analysis. After this is constructed single-copy genes are added to *A *in a way that does not change the DCJ distance (1). This simply involves inserting in *A *each run of single-copy genes next to one of its adjacent (in *T*) double-copy genes *g*, and inserting the same run of single-copy genes next to the duplicate of *g *as well. Sometimes both copies of *g *have adjacent single-copy runs in *T*, due to the process of fractionation. In this case the two single-copy runs must be merged (or *consolidated *[15]). Using present methods, evidence from *R *does not contribute to how this merger proceeds, so that the gene order in this consolidated run may have a large random component. This is particularly true of longer runs, with more than two or three genes.

Adding some randomness to a gene order will tend to create roughly one new rearrangement per added breakpoint [34] and fractionation tends to involve deletions of two or three consecutive WGD paralogs [18], though many of the deletions will be adjacent, creating longer runs of single-copy genes.

This suggests that the distance between *A *and *R *may be exaggerated by a the addition of anywhere from $\frac{1}{4}$*s *to $\frac{1}{2}$*s *on the average for each single-copy run of length *s *for *s *larger than some cutoff value. Therefore, as a crude correction, we deduct from the distance $\frac{1}{3}$*s *for *s *> 3.

**Figure 7:**
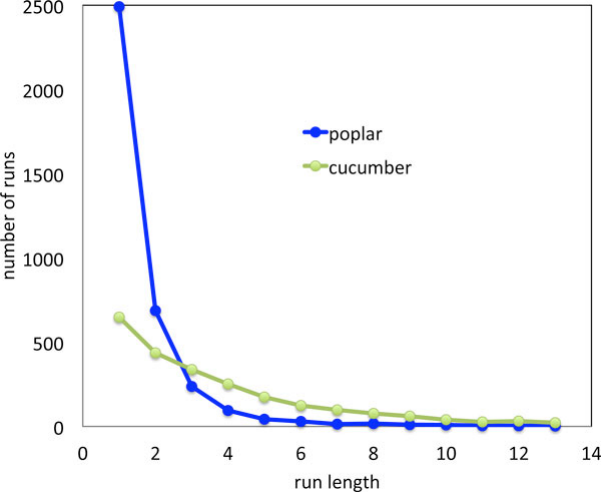
**Single-copy runs in WGD descendants**. Distribution of length of runs of single-copy genes in cucumber and poplar genomes.

The two genomes for which this is pertinent are cucumber and poplar. Figure 7 shows the very different distributions of single-copy run lengths *s *for the two genomes, reflecting the relative recency of the poplar WGD. The distance correction turns out to be 2524 for cucumber but only 536 for poplar. The distances portrayed in the next section incorporate these corrections.

### The Malpighiales

**Figure 8:**
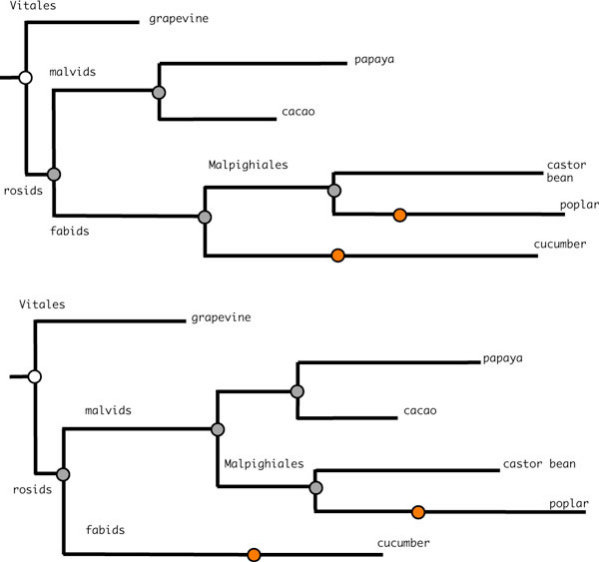
**Positioning the Malpighiales**. Competing hypotheses for the phylogenetic assignment of the Malpighiales, with branch lengths proportional to genomic distances, following the reconstruction of the ancestral genomes with PATHGROUPS. Red nodes indicate WGD event.

In the process of reconstructing the ancestors, we can also graphically demonstrate the great spread in genome rearrangement rates among the species studied, in particular the well-known conservatism of the grapevine genome, as illustrated by the branch lengths in Figure 8.

It has been suggested recently that the order Malpighiales should be assigned to the malvids rather than the fabids [35]. In our results, the tree supporting this suggestion is indeed more parsimonious than the more traditional one. However, based on the limited number of genomes at our disposal, this is not conclusive.

### Properties of the solution as a function of synteny block size

**Figure 9:**
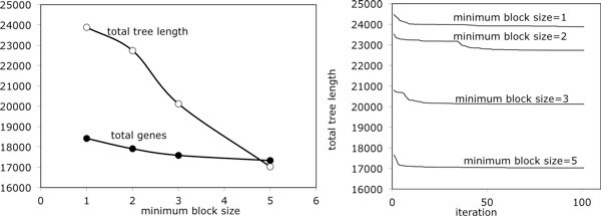
**Role of minimum block length parameter**. Left: Effect of minimum block size on number of orthology sets and total tree length. Right: Convergence behaviour as a function of minimum block size.

To construct the trees in Figure 8, from the 15 pairwise comparisons of the gene orders of the six dicot genomes, we identified some 18,000 sets of orthologs using SYNMAP and the OMG procedure. This varied surprisingly little as the minimum size for a synteny block was set to 1, 2, 3 or 5, as in Figure 9. On the other hand, the total tree length was quite sensitive to minimum synteny block size. This can be interpreted in terms of risky orthology identifications for small block sizes.

Of the 18,000 orthology sets, the number of genes considered on each branch ranged from 12,000 to 15,000. When the minimum block size is 5, the typical branch length over the 11 branches of the tree (including one branch from each WGD descendant to its perfectly doubled ancestor plus one from that ancestor to a speciation node) is about 1600, so that $\frac{d}{n}$ is around 0.12, a low value for which simulations have shown PATHGROUPS to be rather accurate, at least in the equal genomes context [11].

Figure 9 shows the convergence behaviour as the set of medians algorithms is repeated at each ancestral node. Each iteration required about 8 minutes on a MacBook.

### Block validation

To what extent do the synteny blocks output by SYNMAP for a pair of genomes appear in the reconstructed ancestors on the path between these two genomes in the phylogeny? Answering this in a positive way could validate the notion of syntenic conservation implicit in the block construction. If, however, the ancestors did not reflect the pairwise block construction due to conflicting homology structure among other descendants of the same ancestors, we would be forced to discount the pairwise syntenies as artifactual.

Since our reconstructed ancestral genomes are not in the curated COGE database (and are lacking the DNA sequence version required of items in the database), we cannot use SYNMAP to construct synteny blocks between modern and ancestor genomes. We can only see if the genes in the original pairwise syntenies tend to be colinear as well in the ancestor.

On the path connecting grapevine to cacao in the phylogeny in Figure 1, there are two ancestors, the malvid ancestor and the rosid ancestor. There are 308 syntenic blocks containing at least 5 genes in the output of SYNMAP. A total of 11,229 genes are involved, of which 10,872 and 10,848 (97%) are inferred to be in the malvid and rosid ancestor respectively.

Table 1 shows that in each ancestor, roughly half of the blocks appear intact. This is indicated by the fact there are zero syntenic breaks in these blocks (no rearrangement breakpoints) and the average amount of relative movement of adjacent genes within these blocks is less than one gene to the left or right of its original position almost all of the time. Most of the other blocks are affected by one or two breaks, largely because the ancestors can be reconstructed with confidence by PATHGROUPS only in terms of a few hundred chromosomal fragments rather than intact chromosomes, for reasons given in our detailed presentation of PATHGROUPS above. And it can be seen that the average shuffling of genes within these split blocks is little different from in the intact blocks.

**Table 1:** Integrity of cacao-grapevine syntenic blocks

	malvid ancestor		rosid ancestor	
**synteny breaks**	**number**	**intra-block movement ≤ 1.0)**	**number**	**intra-block movement ≤ 1.0)**

0	140 (45%)	126 (90%)	153 (50%)	146 (95%)
1	66 (21%)	62 (94%)	64(21%)	58 (91%)
2	42 (14%)	39 (93%)	47(15%)	37 (79%)
> 2	60 (19%)	58 (97%)	44(14%)	38 (86%)

## Discussion

We have developed a methodology for reconstructing ancestral gene orders in a phylogenetic tree, minimizing the number of genome rearrangements they imply over the entire tree. The input is the set of synteny blocks produced by SYNMAP for all pairs of genomes. The two steps in this method, OMG and PATHGROUPS, are parameter-free; we argue that the proper moment for entering thresholds and other parameters, as well as resolving paralogy, is in the pairwise synteny construction. Our method rapidly and accurately handles large data sets (tens of thousands of genes per genome, and potentially dozens of genomes), although we have been constrained, for non-technical reasons (i.e., embargoes), to present the case of 6 genomes only. There is no requirement of equal gene complement.

For larger numbers of genomes, the quadratic increase in the number of pairs of genomes would become problematic, but this could be handled by extracting information from SYNMAP only from genomes pairs that are relatively close phylogenetically.

Future work will concentrate first on ways to complete cycles in the breakpoint graph which are currently left as paths, without substantially increasing computational complexity. This will increase the accuracy (optimality) of the results. Second, the incorporation of WGD descendants in the phylogeny will be upgraded to reflect the new unequal gene content techniques, in order to reduce the crude correction terms now associated with single-copy regions. Third, to increase the biological utility of the results, a post-processing component will be added to differentiate regions of confidence in the reconstructed genomes from regions of ambiguity.

## Availability

The PATHGROUPS software, together with sample data, may be downloaded from http://137.122.149.195/IsbraSoftware/smallPhylogenyInDel.html

The OMG software, together with sample data, may be downloaded from http://137.122.149.195/IsbraSoftware/OMGMec.html 

The data used here, as well as other genomic data, and the SynMap software for producing pairwise homology sets are available at http://genomevolution.org/CoGe/OrganismView.pl and http://genomevolution.org/CoGe/SynMap.pl, respectively.

Because of the variety of formats in which genome data are released, and incorporated into COGE, the conversion of several SynMap pairwise homology outputs into a master homology graph, conserving positional (on chromosome, fragment, contig, scaffold, pseudomolecule, etc.) information, at this time still requires short programs or scripts specific to the genomes under study.

## Competing interests

The authors declare that they have no competing interests.

## Authors' contributions

CZ and DS did the research for this project and both wrote the paper.
